# Scottish Medical Schools

**Published:** 1898-09-03

**Authors:** 


University College: Medical School.

### SCOTTISH MEDICAL SCHOOLS.

### EDINBURGH.

Edinburgh University.

School of Medicine of the Royal Colleges, Edinburgh.

Medical College for Women, Edinburgh.

The lectures at all these schools are recognised by the University of Edinburgh. Clinical instruction is given at the Royal Infirmary and other institutions.

GLASGOW.

Glasgow University.

Queen Margaret College (the Women's Department of the University).

St. Mungo's College, Glasgow.

Anderson's College Medical School.

DUNDEE.

University College, the classes at which qualify for the degrees of the University of St. Andrews.

ABERDEEN.

University of Aberdeen.

